# SLC23A2-05 (rs4987219) and KRAS-LCS6 (rs61764370) polymorphisms in patients with squamous cell carcinoma of the head and neck

**DOI:** 10.3892/ol.2014.2029

**Published:** 2014-04-03

**Authors:** MARÍLIA BUENO SANTIAGO, FERNANDO AUGUSTO DE LIMA MARSON, RODRIGO SECOLIN, JOSÉ DIRCEU RIBEIRO, CARMEN SÍLVIA PASSOS LIMA, CARMEN SILVIA BERTUZZO

**Affiliations:** 1 Department of Medical Genetics, Center for Pediatrics Research, CIPED, University of Campinas, University City Zeferino Vaz, Campinas, São Paulo 13083-887, Brazil; 2 Department of Pediatrics, Center for Pediatrics Research, CIPED, University of Campinas, University City Zeferino Vaz, Campinas, São Paulo 13083-887, Brazil; 3 Department of Clinical Medicine, School of Medical Sciences, Faculty of Medical Sciences, University of Campinas, University City Zeferino Vaz, Campinas, São Paulo 13083-887, Brazil

**Keywords:** v-Ki-ras2 Kirsten rat sarcoma viral oncogene homolog, solute carrier family 23 member 2, head and neck cancer, polymorphisms, modifier genes, multifactor dimensionality reduction, permutation test

## Abstract

Cancer is a genetic disease that is highly influenced by environmental factors. To determine the risk factors of squamous cell carcinoma of the head and neck, two polymorphisms, solute carrier family 23 member 2 (SLC23A2-05 [rs4987219]) and v-Ki-ras2 Kirsten rat sarcoma viral oncogene homolog (KRAS)-LCS6 (rs61764370), and environmental factors, including smoking and alcohol consumption, were studied in a population. The present study included 165 males diagnosed with squamous cell carcinoma of the head and neck. The control group consisted of 230 healthy male subjects without cancer or a family history of cancer. The SLC23A2-05 and KRAS-LCS6 polymorphisms were analyzed by polymerase chain reaction followed by enzymatic digestion. All patients and healthy subjects were assessed with regard to their smoking habit and alcohol consumption as these are considered to be risk factors for cancer. The statistical analysis was performed using logistic regression, Fisher’s exact and χ^2^ tests. Additional analyses were performed using the programs, multi-factor dimensionality reduction (MDR; version 2.0) and MDR permutation test (version 0.4.7), which consider all variables as risk factors simultaneously. The results of the present study demonstrate that the SLC23A2-05 and KRAS-LCS6 polymorphisms are not a risk factor for squamous cell carcinoma of the head and neck. In the same samples, the association of alcohol consumption (P<0.001) and smoking habit (P<0.001) with cancer presence was positive when each variable was considered individually. Concerning the environmental factors, a positive association of smoking habit and alcohol consumption with cancer, although not with ethnicity (ratio, 1.0244; testing balance accuracy, 0.8733; P<0.001) was identified using the MDR tool, which analyzed the variables and polymorphism genotypes simultaneously. In conclusion, in the present study, squamous cell carcinoma of the head and neck was highly affected by environmental factors when compared with the affect of SLC23A2-05 and KRAS-LCS6 polymorphisms.

## Introduction

Cancer of the head and neck is associated with high morbidity and mortality rates of patients worldwide, including Brazil, and is a global health concern (1,2). Studies have been conducted to determine the genes and environmental factors that are involved in the etiology and modulation of the presence and severity of head and neck cancer (3–8).

The v-Ki-ras2 Kirsten rat sarcoma viral oncogene homolog (KRAS)-LCS6 (G>T) polymorphism is located in the *KRAS2* gene (region 12p12.1, 6 exons; exon 5 has an alternative splicing site and results in the B isoform, *KRASB*) (9) and appears to reduce the lifespan of head and neck cancer patients. Therefore, this variant may be an indicator for the phenotype or therapeutic response (10).

In 2005, three complementary *RAS* genes, *HRAS*, *KRAS* and *NRAS,* were reported in the 3′untranslated region (UTR) region with the expression regulated by the let-7 microRNA (miRNA). The let-7 miRNA expression was reduced in lung tumor tissue compared with normal tissue, however, the RAS protein expression was greater in lung tumors, which indicates a potential underlying mechanism of let-7 miRNA in cancer (11). Furthermore, miRNAs are significant in gene regulation and altered gene expression in tumors (12). The let-7 miRNA is complementary to the *KRAS* 3′UTR polymorphism as a possible risk factor for cancer (13) and the let-7 family may be involved in *KRAS* gene regulation (14).

In small cell lung cancer, smokers aged >40 years with the *KRAS* let-7 (KRAS-LCS6) variant possessed an increased risk of disease. Furthermore, the KRAS-LCS6 variant allele is associated with a high *KRAS* expression and decreased levels of let-7 (13). In this context, the interaction between the let-7 miRNA and *KRAS* gene as a control mechanism via the KRAS variants has been described. Following growth factor binding, cascade activation occurs, which can be blocked via binding to let-7 miRNA. Thus, when the binding between the *KRAS* gene and let-7 miRNA occurs, the cascade is interrupted and the translation of the genes, which are responsible for cell multiplication, remains blocked. When the let-7 miRNA concentration decreases, the cascade is released and translation occurs. However, the KRAS-LCS6 allele and let-7 miRNA binding is hampered, therefore the cascade is released, and translation is facilitated. This observation may be in response to the reduced survival rate of individuals with head and neck cancer, and the KRAS-LCS6 allele variant (10). In addition, the low level of let-7a-2 is associated with a poor survival rate of patients with lung adenocarcinoma (15).

Another polymorphism is the solute carrier family 23 member 2 (SLC23A2)-05 (variation G to C 8 in intron 8, position 48,263), which is localized in the SLC23A2 (nucleobase transporter) member 2, region 20p13-p12. The sodium-dependent SLC23A2 protein is responsible for the transport of vitamin C (16) and is considered to be a risk factor of head and neck cancer in individuals carrying human papillomavirus 16 (HPV16) (17). The SLC23A2-05 polymorphism has been associated with squamous cell carcinoma of the head and neck. However, the association between the SLC23A2-05 variation and positive HPV, as a risk factor for squamous cell carcinoma of the head and neck, is ambiguous. Patients with positive HPV have an increased risk, while those with negative HPV have a reduced risk for cancer (18).

In this context, the objective of the present study was to evaluate the prevalence of the KRAS-LCS6 and SLC23A2-05 polymorphisms in patients with squamous cell carcinoma of the head and neck, and to verify the significance of the polymorphisms on the involvement and severity of the disease via an association study.

## Materials and methods

### Patients with squamous cell carcinoma of the head and neck

A cross-sectional study was performed according to a case-control model. The study was conducted by comparing the case (squamous cell carcinoma of the head and neck diagnosed by clinical and laboratory variables) and control (subjects were paired by gender and age to the case subjects) groups. A negative family history of cancer was considered to be an inclusion criterion.

There were 165 males diagnosed with squamous cell carcinoma of the head and neck that were treated at the Clinical Oncology and Otorhinolaryngology, Faculty of Medical Sciences, State University of Campinas (Campinas, São Paulo, Brazil). The DNA samples were stored at the Laboratory of Cancer Genetics (State University of Campinas, Campinas, Brazil). The control group comprised of 230 healthy male subjects.

The present study was approved by the Ethics committee of the State University of Campinas (no. 873/2007; Campinas, Brazil) and written informed consent for participation in the study was obtained from the subjects.

### DNA extraction from blood

The extraction of DNA from peripheral blood leukocytes was performed according to standard protocol for the phenol-chloroform method (19), with specific modifications. The DNA concentration that was used for analysis was 50 ng/ml, evaluated using a NanoVue™ Spectrophotometer (GE Healthcare Biosciences, Pittsburgh, PA, USA).

### Clinical variables

The clinical variables analyzed in the samples were as follows: Cancer presence (control or case group), ethnicity (Caucasian or not), fatality, smoking habit (smoker, non-smoker or ex-smoker; smokers were defined as individuals who smoked until the point of diagnosis and ex-smokers were those that had stopped smoking ≤5 years prior to diagnosis), alcohol consumption (alcoholic, teetotal or ex-alcoholic; an alcoholic was defined as a patient who had used alcohol until the point of diagnosis, teetotal were patients who had never consumed alcohol and ex-alcoholics had stopped prior to the diagnosis), age at diagnosis, tumor-node-metastasis (TNM) staging system (I, II, III and IV), degree of cancer differentiation (poor, moderate or well) and localization. For the control group, the variables considered were smoker/alcoholic (smoked/consumed alcohol up to the time of last visit), ex-smoker/ex-alcoholic (stopped smoking/consuming alcohol prior to the last visit) and non-smoker/teetotal.

Medical specialists assessed all of the variables, with special consideration given to TNM stage and the adenocarcinoma differentiation degree. The evaluated variables were normalized according to the literature (20).

### SLC23A2-05 (rs4987219) and KRAS-LCS6 (rs61764370) polymorphism analysis

The polymorphism analysis was by polymerase chain reaction (PCR) and enzymatic digestion. To determine the KRAS-LCS6 and SLC23A2-05 polymorphism genotypes the following PCR conditions were used: Bidistilled water, 10× Taq Buffer with KCl, MgCl_2_ (25 mM), dNTP (25 mM for each nitrogenous base), primers (0.2 pmol of sense and antisense primers), Taq polymerase (5 units) and genomic DNA (50 ng/ml). The primers used were as follows: Sense, 5′-GGT GTC AGA GTC TCG CTC TT-3′ and antisense, 5′-GGG TCG TAT ACC AAA GGC CTT AG-3′ for the KRAS-LCS6 polymorphism (to amplify a fragment of 420 bp); sense, 5′-AAA TGC TCT GGG CAA CCT TA-3′ and antisense, 5′-CCC CCA GGA CAT CGA CAA-3′ for the SLC23A2-05 polymorphism (to amplify a fragment of 385 bp).

The annealing temperature was 64.6 and 64.9°C for the KRAS-LCS6 and SLC23A2-05 polymorphisms, respectively, for one minute. The initial denaturation was at 95°C for 5 min, followed by 35 cycles of 94°C for 1 min (with an annealing temperature specific to each polymorphism) and 72°C for 2 min; the final amplification was at 72°C for 7 min.

### Enzymatic digestion

Following PCR, the enzymatic digestion was performed with the *Hinf I* enzyme (New England BioLabs, Inc., Ipswich, MA, USA) for the KRAS-LCS6 polymorphism and with *Tai I* (New England BioLabs, Inc.,) for the SLC23A2-05 polymorphism, at 37°C for 14 h according to the manufacturer’s instructions.

The reaction was analyzed on a polyacrylamide gel (12%) with a voltage of 180 V for 4 h. The gel was stained in ethidium bromide solution and visualized on a Typhoon™ scanner (GE Healthcare, Wisconsin, WI, USA).

According to the fragments observed, the genotypes were identified as follows: i) KRAS-LCS6 polymorphism; TT (8+80+135+197 bp), TG (8+80+135+197+332 bp), and GG (8+80+332 bp) and ii) SLC23A2-05 polymorphism; CC (385 bp), CG (128+270 bp) and GG (128+270+385 bp).

### Statistical analyses

The mean age difference between the case and control groups was evaluated by Student’s t-test using the R program (21). The Hardy-Weinberg equilibrium for genotypes was evaluated by the Haploview program (22), and P<0.05 was considered to indicate a statistically significant difference.

Genotypic and allelic association calculations were performed by logistic regression using the logistf function environment in the R program (21), considering each polymorphism individually as a risk factor for a more severe TNM staging as well as for the presence of cancer.

To associate cancer presence with smoking habit, alcohol consumption and ethnicity as risk factors, the Statistical Package for the Social Sciences version 17 (SPSS Inc., Chicago, IL, USA) (23) was used, incorporating Fisher’s exact test and the χ^2^ test. Using the same program and tests, the association between the polymorphisms, and tumor localization and differentiation degree was assessed. A final analysis was performed concerning the combination of the two polymorphisms, KRAS-LCS6 and SLC23A2-05, and the presence of cancer.

Extra data was obtained using the multi-factor dimensionality reduction (MDR) version 2.0 and MDR permutation test (PT) version 0.4.7 programs. The two programs were used in order to evaluate the genetic interaction between the polymorphisms in the samples. The MDR model is non-parametric and genetic model-free data mining for the identification of non-linear interactions among genetic and environmental attributes (24–26). To adjust the results for multiple comparisons, an MDRPT, which calculated 100,000 permutations was performed in the samples.

The statistical power of the samples was estimated using GPower 3.1.6 (27), to detect associations between the phenotype and polymorphisms. The statistical power of the samples in the present study was 86.6%.

## Results

### Population characteristics

In the case group (mean age, 58.53±9.82 years) 100 patients were diagnosed with laryngeal cancer, and 65 were diagnosed with throat (larynx and pharynx) cancer. With regard to smoking habit; 76.98, 21.21 and 1.81% of the subjects were smokers, ex-smokers and non-smokers, respectively, and for alcohol consumption; 55.76, 30.91 and 13.33% were alcoholics, ex-alcoholics or teetotal, respectively.

In the control group (mean age, 57.12±10.27 years), the smoking habit group comprised of 21.61, 41.71 and 36.68% smokers, ex-smokers and non-smokers, respectively, and for alcohol consumption, 9.55, 27.64 and 62.81% were alcoholics, ex-alcoholics or teetotal, respectively. Alcohol consumption was analyzed in 199 control subjects.

### Analysis of the KRAS-LCS6 and SLC23A2-05 polymorphisms

In the KRAS-LCS6 polymorphism case group, 135 (81.82%) patients exhibited the TT genotype, 29 (17.58%) exhibited the TG genotype and 1 (0.61%) exhibited the GG genotype. In the control group, 187 (81.30%) exhibited the TT genotype, 41 (17.83%) exhibited the GT genotype and 2 (0.87%) exhibited the GG genotype. In the case group the allele frequency was 0.91 and 0.09, for the T and G allele, respectively. In the control group the allele frequency was 0.90 and 0.10, for the T and G allele, respectively.

In the case group for the SLC23A2-05 polymorphism, 35 (21.21%) patients exhibited the CC genotype, 84 (50.91%) CG and 46 (27.88%) GG. In the control group, 43 (18.69%) exhibited the CC genotype, 132 (57.39%) CG and 55 (23.91%) GG. In the case and control groups the allele frequency was 0.47 and 0.53, for the C and G allele respectively.

The KRAS-LCS6 polymorphism was in Hardy-Weinberg equilibrium in the case and control groups: P=0.9899 and P=0.9999, respectively; as were the SLC23A2-05 polymorphisms in the case and control groups; P=0.9232 and P=0.0332, respectively (data not shown).

In Table I the association of alcohol consumption (P<0.001), smoking habit (P<0.001) and ethnicity (P=0.1337) with cancer diagnosis is described. In Table II, the association between the KRAS-LCS6 and SLC23A2-05 polymorphisms, and the degree of cancer differentiation (KRAS-LCS6, P=0.140; and SLC23A2-05, P=0.186) and cancer location (KRAS-LCS6, P=0.640; and SLC23A2-05, P=0.447) is shown. The association of the KRAS-LCS6 and SLC23A2-05 polymorphisms with cancer presence is demonstrated in Table III and in Table IV, the correlation between the two polymorphs and TNM staging is shown; P>0.05 for all values.

**Figure 1:**
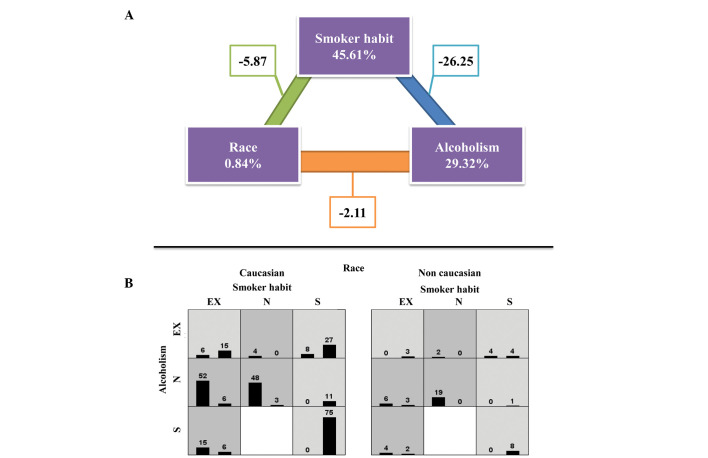
Interaction of the environmental factors (ethnicity, smoking habit and alcohol consumption) and squamous cell carcinoma of the head and neck using multi-factor dimensionality reduction (MDR) and MDR permutation tests. (A) Percentage of the association between the environmental factors. (B) Data distribution. The squares in the figure represent the environmental combination; left bar, controls; right bar, patients. Smoking habit: EX, ex-smoker; N, non-smoker; S, smoker. Alcohol consumption: EX, ex-alcoholic; N, teetotal; S, alcoholic.

The combination of the KRAS-LCS6 and SLC23A2-05 polymorphisms with cancer presence is shown in Table V (P=0.646) and the sample distribution is shown in Fig. 1.

**Figure 2:**
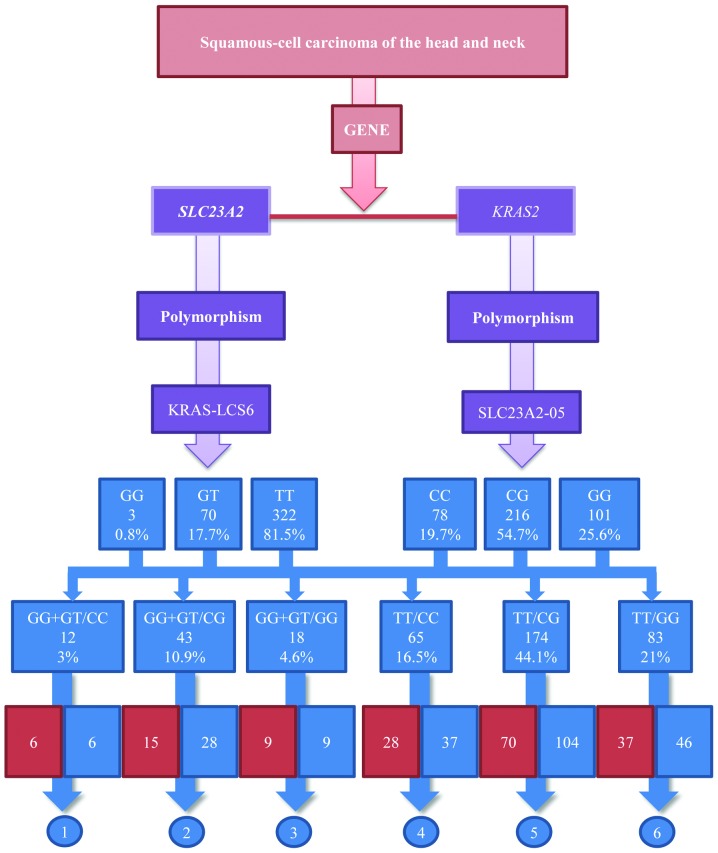
Genotype distribution of KRAS-LCS6 and SLC23A2-05 polymorphisms, and the combination between the polymorphisms. χ^2^ test was performed. 1. OR, 1.408 (CI, 0.422–4.693); 2. OR, 0.722 (CI, 0.364–1.392); 3. OR, 1.415 (CI, 0.534–3.754); 4. OR, 1.066 (CI, 0.618–1.826); 5. OR, 0.893 (CI, 0.595–1.337); 6. OR, 1.156 (CI, 0.796–1.885). *SLC23A2*, solute carrier family 23 member 2; *KRAS*, v-Ki-ras2 Kirsten rat sarcoma viral oncogene homolog; G, guanine; T, thymine; C, cytosine; OR, odds ratio, CI, confidence interval.

The MDR program analysis (Fig. 2) did not identify any statistically significant association between cancer and the KRAS-LCS6 and SLC23A2-05 polymorphisms (ratio, 0.7174; testing balanced accuracy, 0.5157; P=0.4090). For the environmental factors, there was an association between smoking habit and alcohol consumption with cancer; however, this was not identified with ethnicity (ratio, 1.0244; testing balanced accuracy, 0.8733; P=0.0000–0.0010).

## Discussion

Cancer is a complex disease and is a significant health problem. Cancer is a genetic disease, which is highly influenced by environmental factors. Determining the risk factors, including genes and environmental variables, improves current knowledge regarding cancer, provides improved tools for its diagnosis and treatment, as well as a method to determine the risk within a given population. In the present study, squamous cell carcinoma of the head and neck was analyzed considering two polymorphisms, SLC23A2-05 (rs4987219) and KRAS-LCS6 (rs61764370), and environmental factors (smoking habit and alcohol consumption).

For the KRAS-LCS6 polymorphism, the case and control groups were in Hardy-Weinberg equilibrium. In the literature, there is a hypothesis that the activation of the KRAS pathway may occur in squamous cell carcinomas by the action of the KRAS-LCS6 normal variant, which may therefore be associated with the susceptibility and clinical outcome of cancer (10). The present study did not show an association with the overall risk of squamous cell carcinoma of the head and neck, however, cases with the G allele showed a reduction in survival: Hazard ratio (HR), 1.6 and 95% confidence interval; CI, 1.0–2.5. This risk was higher in oral cavity carcinoma (HR, 2.7; 95% CI, 1.4–5.3). The comparison of allele frequencies between the present study and Christensen *et al* (10) is not possible; in the present study, the polymorphisms were analyzed in blood samples, whereas Christensen *et al* (10) analyzed tumor tissue.

In the present study the G allele occurred with a low frequency, in the case and control groups. Therefore, this allele is not considered to be a risk factor to disease susceptibility. The same was found when the sample was evaluated by cancer location. Furthermore, when correlated with the disease stage, no association was found, which indicates no influence on disease progression. However, in the present study the polymorphism was analyzed in the normal tissue of the case patients. Therefore, the hypothesis that this polymorphism may be significant in tumor development could not be excluded, and further investigation of this polymorphism in tumor tissue is required.

In the present study, the SLC23A2-05 polymorphism in the case and control groups demonstrated the same allele frequency. The allele frequency of C and G were 0.47 and 0.53, respectively. In a previous study, C and G allele frequency in an African-American population was 0.80 and 0.20, and in Caucasians was 0.42 and 0.58, respectively (28). In a cancer study, the G and C allele frequency was 0.59 and 0.41 in cancer patients, and 0.57 and 0.43 in control groups. There was a predominance of Caucasian subjects (92 and 93% in the cancer and case groups, respectively) (17). The data of the present study are comparable to the frequencies that were identified in the Caucasian population.

A study by Chen *et al* (17) evaluated 319 patients with squamous cell carcinoma of the head and neck, and 495 control subjects in association with HPV16 status, citrus consumption and the single nucleotide polymorphism (SNP) panel (SNP500Cancer). The SLC23A2-05 polymorphism plus HPV16 infection were considered to be a risk factor for cancer. Among G allele patients with the SLC23A2-05 polymorphism, the cancer risk in association with HPV16 was 5.0 (95% CI, 3.2–7.8). Among the homozygous variant (CC) subjects, the cancer risk associated with HPV16 was attenuated (odds ratio; OR, 2.8 and 95% CI, 1.2–6.2). In determining if the genotype modifies the interaction between citrus consumption, HPV16 and cancer risk, there was an increased risk of cancer found in individuals with one G allele to SLC23A2-5 polymorphism, HPV16 seropositive and high citrus consumption (OR, 7.4; 95% CI, 3.6–15.1). In the present study the polymorphism was not associated with cancer presence or cancer progression.

In TNM staging, there was no statistically significant association identified with the SLC32A2-05 polymorphism. In this context, these polymorphisms may not be cancer severity modulators. As for the KRAS-LCS6 polymorphism, the SLC32A2-05 polymorphism was analyzed in normal tissue.

Concerning smoking habit and alcohol consumption, there was an association with cancer risk. In this context, the squamous cell carcinoma of the head and neck was influenced by environmental factors, however, there was not a modifier gene associated with the polymorphisms that were investigated in the present study.

In conclusion, squamous cell carcinoma of the head and neck is a significant disease worldwide. Therefore, determining the factors that are associated with the severity and presence of this type of cancer is considered to be important. Globally, smoking habit and alcohol consumption are considered to be risk factors, resulting in individuals exhibiting squamous cell carcinoma of the head and neck. In the present study, the KRAS-LCS6 and SLC32A2-05 polymorphisms were not considered to be cancer risk factors, however, they were analyzed in the blood and not in tumor tissue. Potentially, the observation of these polymorphisms in a localized tumor may provide a significant tool for the detection of cancer. In future studies, the investigation of additional genes and tumor tissue is required to clarify the complex mechanisms associated with cancer, as well as protein quantification to determine additional data regarding cancer severity.

## Figures and Tables

**Table I:** Association between the cancer and control groups with environmental factors; smoking, alcohol consumption and ethnicity.

	Group					
					
Environmental factor	Cancer	Control	Total	P-value	OR	CI (5–95%)
Smoker^a^						
EXS	35	83	118	<0.0001^b^	0.377^b^	0.334–0.600^b^
NS	3	73	76		0.032^b^	0.008–0.093^b^
S	127	43	170		12.02^b^	7.374–19.93^b^
Total	165	199	364			
Alcohol consumption^a^						
EXA	49	55	104	<0.0001^b^	1.115	0.705–1.763
NA	24	125	149		0.102^b^	0.06–0.1702^b^
A	91	19	110		11.72^b^	6.748–21.030^b^
Total	164	199	363			
Ethnicity^c^						
C	143	185	328	0.1337	1.579	0.911–2.790
NC	22	45	67		1.000	
Total	165	230	395			

a χ^2^ test;

b positive P-values and ORs;

c Fisher’s Exact test.

OR, odds ratio; CI, confidence interval; EXS, ex-smoker; NS, non-smoker; S, smoker; EXA, ex-alcoholic; NA, teetotal; A, alcoholic; C, caucasian; NC, not caucasian. With regard to all analyses, α=0.05 was considered to indicate significance.

**Table II:** Association of the KRAS-LCS6 and SLC23A2-05 polymorphisms with location and differentiation degree in patients with squamous cell carcinoma of the head and neck identified by the χ^2^ test.

A, Association of the KRAS-LCS6 and SLC23A2-05 polymorphisms with location														

	KRAS-LCS6				SLC23A2-05									
						
Location	GG + TG	TT	Total	P-value	OR	95% CI	CC	OR (95% CI)	CG	OR (95% CI)	GG	OR (95% CI)	Total	P-value
Oral cavity	1	0	1	0.140	-	-	0	-	1	-	0	-	1	0.186
Larynx	16	84	100		0.695	0.310–1.568	16	0.463 (0.214–0.992)^a^	56	1.676 (0.892–3.173)	28	1.015 (0.505–2.067)	100	
Opharynx	13	51	64		1.258	0.553–2.821	19	2.232 (1.042–4.829)^a^	27	0.565 (0.298–1.065)	18	1.02 (0.501–2.052)	64	
Total	30	135	165				35		84		46		165	

B, Association of the KRAS-LCS6 and SLC23A2-05 polymorphisms with degree of differentiation														

	KRAS-LCS6				SLC23A2-05									
						
Degree of differentiation	GG + TG	TT	Total	P-value	OR	95% CI	CC	OR (95% CI)	CG	OR (95% CI)	GG	OR (95% CI)	Total	P-value

Well-differentiated	4	16	20	0.640	1.282	0.340–4.052	2	0.345 (0.052–1.381)	10	0.985 (0.376–2.581)	8	1.971 (0.712–5.267)	20	
Moderately-differentiated	19	105	124		0.623	0.238–1.751	29	1.27 (0.488–3.687)	63	1.101 (0.496–2.453)	32	0.732 (0.313–1.781)	124	
Non-keratinizing	0	1	1		-	-	1	-	0	-	0	-	1	0.447
Poorly-differentiated	3	6	9		2.652	0.51–11.46	3	1.774 (0.346–7.537)	4	0.780 (0.180–3.197)	2	0.758 (0.104–3.573)	9	
Superficially invasive	0	1	1		-	-	0	-	1	-	0	-	1	
Total	26	129	155				35		78		42		155	

a Positive P-values and ORs.

To all analyses, α=0.05 was considered. OR, odds ratio; CI, confidence interval; G, guanine; T, thymine; C, cytosine; SLC23A2, solute carrier family 23 member 2; KRAS, v-Ki-ras2 Kirsten rat sarcoma viral oncogene homolog.

**Table III:** Association between the KRAS-LCS6 and SLC23A2-05 polymorphisms as a risk factor for squamous cell carcinoma of the head and neck as identified by regression analysis.

A, KRAS-LCS6 polymorphism						

	Patients, n		Frequency			
				
Feature	Case group	Control group	Case group (%)	Control group (%)	P-value	OR (95% CI)
Genotype						
GG	1	2	0.61	0.87	0.8597	0.83 (0.08–8.70)
TG	29	41	17.57	17.83	0.9603	0.99 (0.58–1.67)
TT	135	187	81.81	81.30	0.9080	1.03 (0.62–1.73)
Allele						
G	31	45	9.39	9.78	0.9080	0.97 (0.58–1.63)
T	299	415	90.61	90.22	0.8597	1.20 (0.11–12.53)

B, SLC23A2-05 polymorphism						

	Patients, n		Frequency			
				
Feature	Case group	Control group	Case group (%)	Control group (%)	P-value	OR (95% CI)

Genotype						
CC	35	43	21.21	18.69	0.5304	1.17 (0.71–1.93)
CG	84	132	50.90	57.39	0.2026	0.77 (0.52–1.15)
GG	46	55	27.88	23.91	0.3711	1.23 (0.78–1.94)
Allele						
C	154	218	46.67	47.39	0.3711	0.81 (0.52–1.28)
G	176	242	53.33	52.61	0.5304	0.85 (0.52–1.40)

KRAS, v-Ki-ras2 Kirsten rat sarcoma viral oncogene homolog; SLC23A2, solute carrier family 23 member 2; OR, odds ratio; CI, confidence interval; G, guanine; T, thymine; C, cytosine. With regard to all analyses, α=0.05 was considered to indicate significance.

**Table IV:** TNM staging and correlation with the KRAS-LCS6 and SLC23A2-05 polymorphisms in patients with squamous cell carcinoma of the head and neck as identified by regression analysis.

A, *KRAS-LCS6* gene								

	Stage I		Stage II		Stage III		Stage IV	
				
Feature	P-value	OR (95% CI)	P-value	OR (95% CI)	P-value	OR (95% CI)	P-value	OR (95% CI)
Genotype								
GG	0.7263	1.79 (0.04–75.55)	0.5613	2.77 (0.06–118.05)	0.9512	1.10 (0.03–46.06)	0.6419	1.64 (0.16–17.23)
TG	0.2017	1.85 (0.74–4.65)	0.4044	1.64 (0.53–5.13)	0.2259	0.55 (0.19–1.56)	0.9559	1.02 (0.53–1.95)
TT	0.2445	0.57 (0.23–1.43)	0.4586	0.64 (0.21–2.01)	0.1810	1.93 (0.68–5.48)	0.9073	0.96 (0.51–1.82)
Allele								
G	0.2445	1.75 (0.70–4.37)	0.4586	1.55 (0.50–4.83)	0.1810	0.52 (0.18–1.47)	0.9073	1.04 (0.55–1.96)
T	0.7263	0.56 (0.01–23.52)	0.5613	0.36 (0.01–15.39)	0.9512	0.91 (0.02–37.98)	0.6419	0.61 (0.06–6.39)

B, *SLC23A2-05* gene								

	Stage I		Stage II		Stage III		Stage IV	
				
Feature	P-value	OR (95% CI)	P-value	OR (95% CI)	P-value	OR (95% CI)	P-value	OR (95% CI)

Genotype								
CC	0.0627	0.26 (0.05–1.44)	0.8598	1.12 (0.33–3.83)	0.3675	1.44 (0.66–3.13)	0.2147	1.46 (0.81–2.64)
CG	0.5475	1.29 (0.56–3.00)	0.9055	0.94 (0.35–2.55)	0.6492	0.86 (0.44–1.66)	0.0566	0.62 (0.37–1.02)
GG	0.3456	1.54 (0.64–3.70)	0.8220	1.14 (0.37–3.51)	0.8431	0.92 (0.42–2.03)	0.2859	1.36 (0.78–2.36)
Allele								
C	0.3456	0.65 (0.27–1.57)	0.8220	0.88 (0.28–2.71)	0.8431	1.08 (0.49–2.38)	0.2859	0.74 (0.42–1.29)
G	0.0627	3.79 (0.69–20.73)	0.8598	0.89 (0.26–3.07)	0.3675	0.70 (0.32–1.52)	0.2147	0.69 (0.38–1.24)

TNM, tumor-node-metastasis; KRAS, v-Ki-ras2 Kirsten rat sarcoma viral oncogene homolog; SLC23A2, solute carrier family 23 member 2; OR, odds ratio; CI, confidence interval; G, guanine; T, thymine; C, cytosine. With regard to all analyses, α=0.05 was considered to indicate significance.

**Table V:** Combination of the KRAS-LCS6 and SLC23A2-05 polymorphisms and the associated risk of squamous cell carcinoma of the head and neck as determined by the χ^2^ test.

KRAS-LCS6 and SLC23A2-05 polymorphisms	Cancer group	Control group	Total	P-value^a^	OR	95% CI
Combination code						
1	6	6	12	0.826	1.408	0.422–4.693
2	15	28	43		0.722	0.364–1.392
3	9	9	18		1.415	0.534–3.754
4	28	37	65		1.066	0.618–1.826
5	70	104	174		0.893	0.595–1.337
6	37	46	83		1.156	0.796–1.885
Total	165	230	395			

a P-value for haplotype analysis.

Combination code: 1, GG+GT/CC; 2, GG+GT/CG; 3, GG+GT/GG; 4, TT/CC; 5, TT/CG; 6, TT/GG; where KRAS-LCS6 genotype/SLC23A2-05 genotype, respectively. KRAS, v-Ki-ras2 Kirsten rat sarcoma viral oncogene homolog; SLC23A2, solute carrier family 23 member 2; OR, odds ratio; CI, confidence interval; G, guanine; T, thymine; C, cytosine.
